# Patients at high risk of suicide before and during a COVID-19 lockdown: ecological momentary assessment study

**DOI:** 10.1192/bjo.2021.43

**Published:** 2021-04-16

**Authors:** Aurora Cobo, Alejandro Porras-Segovia, María Mercedes Pérez-Rodríguez, Antonio Artés-Rodríguez, Maria Luisa Barrigón, Philippe Courtet, Enrique Baca-García

**Affiliations:** Department of Signal Theory, Universidad Carlos III de Madrid, Spain; and Instituto de Investigación Sanitaria Gregorio Marañón, Spain; Instituto de Investigación Sanitaria Fundación Jiménez Díaz, Spain; and Department of Psychiatry, Hospital Universitario Rey Juan Carlos, Spain; Icahn School of Medicine at Mount Sinai, USA; Department of Signal Theory, Universidad Carlos III de Madrid, Spain; and Instituto de Investigación Sanitaria Gregorio Marañón, Spain; Instituto de Investigación Sanitaria Fundación Jiménez Díaz, Spain; Universidad Autónoma de Madrid, Spain; and Department of Psychiatry, Hospital Universitario Fundación Jiménez Díaz, Spain; University of Montpellier & INSERM u1061, France; Instituto de Investigación Sanitaria Fundación Jiménez Díaz, Spain; Department of Psychiatry, Hospital Universitario Rey Juan Carlos, Spain; Universidad Autónoma de Madrid, Spain; Department of Psychiatry, Hospital Universitario Fundación Jiménez Díaz, Spain; Nimes University Hospital, France; CIBERSAM, Spain; Department of Psychiatry, Hospital Universitario Infanta Elena, Spain; Department of Psychiatry, Hospital Universitario Central de Villalba, Spain; and Universidad Católica del Maule, Chile

**Keywords:** Suicide, suicide attempt, COVID-19, ecological momentary assessment, machine learning

## Abstract

The coronavirus disease 2019 (COVID-19) outbreak may have affected the mental health of patients at high risk of suicide. In this study we explored the wish to die and other suicide risk factors using smartphone-based ecological momentary assessment (EMA) in patients with a history of suicidal thoughts and behaviour. Contrary to our expectations we found a decrease in the wish to die during lockdown. This is consistent with previous studies showing that suicide rates decrease during periods of social emergency. Smartphone-based EMA can allow us to remotely assess patients and overcome the physical barriers imposed by lockdown.

Psychiatric patients are particularly vulnerable to the psychological impact of the coronavirus disease 2019 (COVID-19) outbreak. Social distancing and lockdown measures result in multiple stressors known to increase risk for suicide, including social isolation, financial stress, decreased access to mental healthcare and medical comorbidities.^1^ Research on the mental health consequences of this crisis is considered a priority.^2^ However, quarantine has interfered with face-to-face research. Mobile technology applied to health – known as mobile health or m-Health – can overcome these barriers. In this study we use smartphone-based ecological momentary assessment (EMA) to explore the impact of COVID-19 social distancing and lockdown measures on suicide risk, in a sample of psychiatric patients at high risk for suicide.

## Method

The authors assert that all procedures contributing to this work comply with the ethical standards of the relevant national and institutional committees on human experimentation and with the Helsinki Declaration of 1975, as revised in 2008. All procedures involving human patients were approved by the Ethics Committee of the University Hospital Fundación Jiménez Díaz. All participants provided written informed consent to participate in the study.

### Participants and procedures

Using EMA, we prospectively assessed 36 adult patients, who were being treated at our suicide prevention out-patient clinic because of a high risk of suicide. EMA was delivered using the MEmind smartphone app, which is available for both Android and iOS operating systems. EMA questions were announced as push notifications on users’ screens. A detailed description of the MEmind app has been published elsewhere.^3,4^ Participants were recruited from an ongoing multisite study examining longitudinal risk factors for suicide (SmartCrisis^4^).

Patients were included in the study if they had a history of at least one suicide attempt or an emergency department visit because of suicidal ideation. Written informed consent was obtained from all patients. Pseudonymization of the participants’ personal data was employed, by using a unique identification code for each participant. The follow-up period was divided into: (a) pre-lockdown: 1 October 2019 to 13 March 2020 (before the implementation of Covid-19 lockdown measures); and (b) lockdown: 14 March 14 April 2020.

At baseline and at follow-up, patients were administered the Columbia Suicide Severity Rating Scale (CSSRS).^5^ To safeguard the well-being of our patients, upon detecting an alarming level of suicidal ideation (threshold was established at CSSRS suicidal ideation subscale score ≥ 4), their attending psychiatrist was informed, and it was suggested to patients that they attend the emergency department.

### The EMA questionnaire

**Fig. 1:**
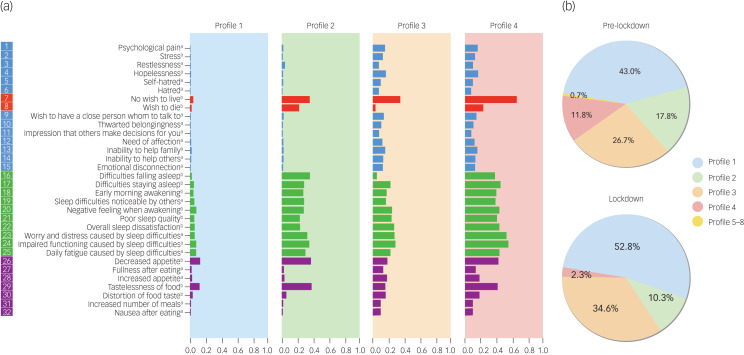
(a) Suicide risk features identified using the Indian buffet process. Vertical axis: variables. Horizontal axis: probability of scoring positive on each of the variables. (b) Distribution of features before and during lockdown. Pre-lockdown: 1 October 2019 to 13 March 2020 (before the implementation of COVID-19 lockdown measures). Lockdown: 14 March to 14 April 2020. In (a) assessment frequency: a. at least once every 2 weeks during the first month and at least once every 6 weeks afterwards; b. at least twice per week during the first month and at least once per week afterwards.

The EMA questionnaire consists of 32 questions grouped into four areas: wish to die/wish to live (2 questions), sleep (10 questions), negative feelings (13 questions) and appetite (7 questions). Questions were based on the Salzburg Suicide Process Questionnaire.^6^ Supplementary Table 1 available at https://doi.org/10.1192/bjo.2021.43 shows all the questions and their scoring. The MEmind EMA questionnaire has shown good acceptability in preliminary studies.^7^ As constant repetition of questions can place a significant burden on the user, we have incorporated a turn-over system for questions. Out of the pool of 32 questions participants were asked two to four random questions every day, at random times from 10.00 to 22.00 h. Figure 1 shows the variables explored in the EMA questionnaire and the frequency with which the questions were asked.

### Statistical analysis

We used a machine learning technique, the Indian buffet process (IBP).^8^ The IBP is a non-parametric Bayesian method that reveals latent features through a sparse analysis. Sparsity is defined by the fact that only some of the datapoints will offer discriminant information. Using this method, we can overcome the missing data caused by the turn-over system of the questions. The features revealed by the IBP are supravariables formed by the grouping of variables, that is: sets of variables that tend to adopt certain abnormal values in the same time frame. They are dynamic, and the same person may have different features over time.

The values of each of the 32 questions were standardised so that all were expressed from 0 to 1 and the highest value would always express a worse state of mental health.

We compared individual suicide risk features before and during the lockdown.

## Results

Mean age of the participants was 41.7 years (s.e. = 16.3). The majority of the participants were women (n = 31; 86.1%). The most common psychiatric diagnosis was mood disorders (n = 21; 58.3%). The mean number of previous suicide attempts was 1.1 (s.e. = 0.2)

We identified four suicide risk features that accounted for more than 99.5% of the participants’ responses (see Fig. 1(a)). Profile 1 is characterised by low values (i.e. low probability of scoring positive) across all 32 suicide risk factors. Profiles 2 and 4 are characterised by a high desire for death, lack of wish to live, decreased appetite and tastelessness of food, and sleep problems; Profile 4 also shows high values for negative emotions. Profile 3 is characterised by lower desire for death, and lower appetite and sleep symptoms, with high values of negative emotions.

Before quarantine, the most prevalent feature was Profile 1, with 43.0%. That is, of the 960 responses before quarantine, 43.0% were grouped in Profile 1. The second most prevalent was Profile 3 (26.7%), followed by Profile 2 (17.8%). Profile 4 accounted for 11.8% of the responses and the remaining profiles (5 to 8) accounted for 0.7% (95% CI 0.3–1.5%) of the responses.

During the quarantine, the dominant feature continued to be Profile 1 (52.8%). That is, of the 214 responses, 52.8% were grouped around Profile 1. This represents a 22.8% increase. The second most common profile was still Profile 3, with 34.6%. This represents a 29.6% increase. Profile 2 fell to 10.3%, a 42.1% decrease, and Profile 4 fell to 2.3%, an 80.1% decrease. The remaining profiles (5–8) were not represented during the quarantine.

Results of the χ^2^-test show there are statistically significant differences before *v*. during lockdown (Profile 1: χ^2^ = 6.38, *P* = 0.012; Profile 2: χ^2^ = 6.69, *P* = 0.010; Profile 3: χ^2^ = 5.04, *P* = 0.025; Profile 4: χ^2^ = 16.20, *P* < 0.001).

## Discussion

Contrary to our expectations, we observed that self-reported suicide risk appeared to decrease during a COVID-19-related lockdown period, in a prospective cohort monitored using smartphone-delivered EMA. Specifically, we found a decrease in the wish to die, and in the rates of appetite and sleep symptoms.

### Strengths and limitations

Strengths of our study include the prospective design and real-time monitoring of dynamic suicide risk using EMA. Our results should be interpreted with caution given the modest sample size. This modest sample size may be the reason why we have found an uneven gender distribution, with over 85% of patients being women. However, in a prior EMA study by our research group we also found a predominance of women in the sample.^7^ Another potential limitation is that we did not ask directly about suicide intent but employed the indirect measure ‘wish to die’. However, a recent systematic review and meta-analysis exploring passive suicide ideation found that it was highly similar to active suicide ideation and that it was strongly associated with suicide attempts.^9^ Also, the observation period before lockdown was longer than during lockdown. Finally, the length of the follow-up period was not uniform across the sample.

### Comparison with findings from other studies

Other studies have also found a decrease in suicidal ideation as a result of COVID-19-related measures. For instance, a recent study showed that internet search queries related to suicide decreased after the USA issued stay-at home-orders.^10^ Although it may seem surprising that suicidal ideation decreases, it is actually consistent with some previous studies showing a drop in suicide rates during periods of social emergency, such as wartime or terrorist attacks.^11,12^ However, there is also evidence indicating that this decrease may be just temporary: the study by Batty et al (2018)^13^ shows that, although there is a decrease in suicidal behaviour during wartime, just after wars end, suicidal behaviour increases to levels higher than those observed before the war. Thus, during the post-war period, the harmful effects of conflict on an individual's mental health become apparent. In the same way, the possibility exists that there will be an increase in suicidal ideation and behaviour above the expected level once the acute COVID-19 crisis ceases. We must be prepared for this contingency.

### Implications

Continuity of care has been affected by the COVID-19 crisis. In order to minimise the risk of contagion, non-urgent face-to-face consultations have been discontinued in many countries, including Spain. Telemedicine allows us to continue to provide mental healthcare services to our patients. New technologies are already being used to preserve people's mental healthcare during the COVID-19 crisis, for example in the form of online services.^14^

Ensuring access to adequate mental healthcare for vulnerable populations, such as psychiatric patients at high risk for suicide, should remain a priority during times of social emergencies. Smartphone-based monitoring can be used to monitor high-risk populations during social distancing and lockdown periods.

## Data Availability

The data that support the findings of this study are available on request from the corresponding author, E.B.-G..

## Supplementary material

For supplementary material accompanying this paper visit http://dx.doi.org/10.1192/bjo.2021.43. click here to view supplementary material
