# Supplementary material for: Patients at high risk of suicide before and during a COVID-19 lockdown: ecological momentary assessment study
Source: BJPsych Open. 2021 Apr 16;7(3):e82. doi: 10.1192/bjo.2021.43 (PMC8060530; doi:10.1192/bjo.2021.43)
Supplement: Supplementary file 1 [file S2056472421000430sup001.docx]

| **Supplementary table 1**. Ecological Momentary Assessment questionnaire | | | | | |
| --- | --- | --- | --- | --- | --- |
| Number | Area | Question | Minimum value | Maximum value | Scoring |
| 1 | Negative feelings | Today I feel psychological pain | No pain | Maximum pain | 1-7 |
| 2 | Negative feelings | I feel stressed out today (with pressure, overwhelmed) | No stress | Maximum stress | 1-7 |
| 3 | Negative feelings | Today I feel restless (agitated), with the need to keep moving | No restlessness | Maximum restlessness | 1-7 |
| 4 | Negative feelings | Today I feel full of hope | No hope | Maximum hope | 1-7 |
| 5 | Negative feelings | Today I have felt hatred or anger towards myself | No hatred | Maximum hatred | 1-7 |
| 6 | Negative feelings | Today I have felt hatred or anger towards others | No hatred | Maximum hatred | 1-7 |
| 7 | Passive suicidal ideation | Today I feel the wish to live | No wish to live | Maximum wish to live | 1-7 |
| 8 | Passive suicidal ideation | Today I feel the wish to die | No wish to die | Maximum wish to die | 1-7 |
| 9 | Negative feelings | Today I wished I had a trusted person to tell my personal issues | Not at all | Absolutely | 1-7 |
| 10 | Negative feelings | Today I felt like a stranger (out of place) | Not at all | Absolutely | 1-7 |
| 11 | Negative feelings | Today I had the impression that important people around me want to decide for me what I should think and do | Not at all | Absolutely | 1-7 |
| 12 | Negative feelings | Today I have wished to receive more recognition and love from others | Not at all | Absolutely | 1-7 |
| 13 | Negative feelings | Today I believe I have contributed to the well-being of my family/friends | Not at all | Absolutely | 1-7 |
| 14 | Negative feelings | Today I believe I have contributed to the well-being of the people around me | Not at all | Absolutely | 1-7 |
| 15 | Negative feelings | Today I felt disconnected from the rest of the people | Not at all | Absolutely | 1-7 |
| 16 | Sleep problems | Last night I had trouble sleeping | None | Very severe | 0-4 |
| 17 | Sleep problems | Last night I had trouble staying asleep | None | Very severe | 0-4 |
| 18 | Sleep problems | This morning I had trouble waking up early | None | Very severe | 0-4 |
| 19 | Sleep problems | Currently, others think that sleep problems affect my quality of life | Not at all | Absolutely | 1-7 |
| 20 | Sleep problems | Today when I woke up, I felt | Very bad | Very good | 1-7 |
| 21 | Sleep problems | Last night the quality of my sleep was... | Very bad | Very good | 1-7 |
| 22 | Sleep problems | Today I am satisfied with my sleep | Very unsatisfied | Very satisfied | 1-7 |
| 23 | Sleep problems | I am currently worried or stressed about my sleep problems | Not at all | Very much | 0-4 |
| 24 | Sleep problems | Currently my sleep problems are interfering with my daily activity | Not at all | Very much | 0-4 |
| 25 | Sleep problems | Today I feel tired during the day because of my sleep problems | Not at all | Absolutely | 1-7 |
| 26 | Appetite | In the last few days my appetite is... | Very little | Very big | 0-4 |
| 27 | Appetite | In the last days when I eat full after eating... | Only a few bites | Almost never | 0-4 |
| 28 | Appetite | In the last few days I have been hungry | Never | All the time | 0-4 |
| 29 | Appetite | In the last days when I eat, the food tastes... | Very bad | Very good | 0-4 |
| 30 | Appetite | Compared to some years ago, nowadays the food tastes... | Much worse | Much better | 0-4 |
| 31 | Appetite | In the last few days I usually do... | Less than one meal a day | More than three meals a day | 0-4 |
| 32 | Appetite | In the last few days when I eat, I feel sick or nauseous... | Most times | Never | 0-4 |
